# Measurement of American Indian and Alaska Native Racial Identity Among Medical School Applicants, Matriculants, and Graduates, 1996-2017

**DOI:** 10.1001/jamanetworkopen.2020.32550

**Published:** 2021-01-19

**Authors:** Erik Brodt, Steele Valenzuela, Allison Empey, Amanda Bruegl, Dove Spector, Miguel Marino, Patricia A. Carney

**Affiliations:** 1 Department of Family Medicine, Oregon Health & Science University, Portland; 2 Ojibwe, Minnesota; 3 Department of Family Medicine, Oregon Health & Science University, Portland; 4 Omaha Tribe of Nebraska; 5 Department of Pediatrics, Oregon Health & Science University, Portland; 6 Confederated Tribes of Grand Ronde, Oregon; 7 Department of Obstetrics-Gynecology, Oregon Health & Science University, Portland; 8 Oneida and Stockbridge-Munsee Nations, Wisconsin; 9 Department of Family Medicine, Oregon Health & Science University, Portland; 10 Nez Perce Tribe, Idaho; 11 Department of Biostatistics, Oregon Health & Science University, Portland; 12 Department of Family Medicine, Portland State University School of Public Health, Portland, Oregon

## Abstract

**Importance:**

Accurate racial/ethnic identity measurement is needed to understand the effectiveness of outreach, recruitment, and programs to support American Indian and Alaska Native (AIAN) people becoming physicians.

**Objective:**

To examine how changes in race/ethnicity data collection by the American Medical College Application System are associated with trends in applicants, matriculants, and graduates self-reporting as AIAN.

**Design, Setting, and Participants:**

In this cohort study, interrupted time series regression was conducted using data from the American Medical College Application system identifying medical school applicants and graduates between January 1, 1996, and December 31, 2017, who identified as AIAN. The number of students identifying as AIAN was compared before and after the American Medical College Application System changed how it collected race/ethnicity data in 2002. Data analyses were conducted between December 2019 and May 2019.

**Exposures:**

Applicants could select only 1 racial identity from 1996 to 2001 and could select more than 1 racial identity from 2002 to 2017.

**Main Outcomes and Measures:**

Rates of AIAN groups before and after changing how race/ethnicity data were collected. Covariates were age, sex, and Medical College Admission Test scores.

**Results:**

The total number of individuals identifying as AIAN in the study was 8361; the mean (SD) number of applicants per year was 380.0 (89.9) overall: 257.3 (39.6) in 1996 to 2001, with a mean (SD) age of 26.6 (5.5) years and 830 (54.0%) male individuals, and 426.1 (50.1) in 2002 to 2017, with a mean (SD) age of 25.5 (5.6) years and 3441 (50.5%) female individuals. Before the change, there was a decrease of 5% per year (relative rate [RR] of 0.95; 95% CI, 0.91-0.98; *P* < .001) in the rate of AIAN applicants. In 2002, the change in data collection was associated with an immediate 78% relative increase in applicants (RR, 1.78; 95% CI, 1.55-2.06; *P* < .001). From 2002 to 2017 there was a 10% increase in applicants per year (RR, 1.10; 95% CI, 1.06-1.14; *P* < .001). For matriculants, yearly trends indicated a nonsignificant 3% decrease before the change, whereas the change was associated with an immediate 62% relative increase in matriculants (RR, 1.62; 95% CI, 1.35-1.95; *P* < .001), with no difference in trend after the change. For graduates, a nonsignificant yearly decrease of 2% was found in the mean number of graduates before the change, whereas the change was associated with an immediate 94% relative increase (RR, 1.94; 95% CI, 1.57-2.38; *P* < .001), followed by no change in trend after the modification.

**Conclusions and Relevance:**

Changing the method of race/ethnicity data collection captured more AIAN applicants, matriculants, and graduates. Yearly trends indicate concerning although nonsignificant differences after the change for AIAN graduates. These findings should inform diversity efforts.

## Key Points

**Question:**

Are changes in collection of race/ethnicity data in American Medical College Application System surveys in 2002 associated with temporal trends in applicants, matriculants, and graduates who self-reported as American Indian/Alaska Native (AIAN)?

**Findings:**

This cohort study found that, after the change in data collection, the mean number of AIAN applicants increased by 78%; matriculants, 62%; and graduates, 94%. The numbers of AIAN applicants continued to increase at 10% per year following the change, but no significant change in trend was found for matriculants or graduates.

**Meaning:**

These findings suggest that changing the method of race/ethnicity data collection captures more AIAN applicants, matriculants, and graduates, but yearly trends indicate few differences after the change in terms of AIAN graduates.

## Teaser

This cohort study uses interrupted time series regression to assess the association between changes in race/ethnicity data collection in the American Medical College Application System survey and trends in applicants, matriculants, and graduates self-reporting as American Indian and Alaska Native.

## Introduction

Efforts to diversify the physician workforce are crucial to meeting the health care needs in underserved communities across the US. Health disparities are particularly problematic among American Indian and Alaska Native (AIAN) populations, which have been affected by socioeconomic determinants,^1,2,3,4,5^ including higher rates of morbidity, mortality, unmet health care needs and lower health care use compared with other racial/ethnic groups, even when access to care is taken into account.^6^ People of AIAN race/ethnicity are more likely to have overweight or obesity, lack physical activity, smoke, use alcohol, and have cardiovascular disease, mental distress, and associated chronic conditions compared with their White counterparts.^3,5^ These issues are further complicated by ongoing physician workforce shortages. Indian Health Service data from November 2017 indicate that the overall percentage of vacancies for physicians, nurses, nurse practitioners, certified registered nurse anesthetists, certified nurse midwives, physician assistants, dentists, and pharmacists averaged 25% and ranged from 13% to 31%.^7^

American Indian and Alaska Native physicians are more likely to work with AIAN populations than their non-AIAN peers^2^; however, there is a paucity of AIAN people with medical degrees.^8,9,10,11^ According to 2018 data, 2570 (0.3%) of the 918 547 physicians active in the US reported being AIAN alone or in combination with another race.^12^ Unfortunately, the number of US medical school applicants identifying as AIAN only decreased by 32% between 1980 and 2013.^13^ This negative trend appears to be accelerating, with a 70% decrease in AIAN-only applicants and a 63% decrease in AIAN-only matriculants to US medical schools between 1996 and 2016.^13^ These findings suggest that efforts to diversify the medical workforce for AIAN people are lacking, leading to deficiencies in the numbers of AIAN physicians and in racially concordant care.^2^

American Indian and Alaska Native identity has long been a subject of contentious debate among legal entities, policymakers, and others, and it has been redefined many times during the past 150 years.^14^ This contention has placed a substantial burden on both tribal affiliations and American Indian people trying to self-identify within multiple cultural contexts. Although many tribes have adopted the use of blood quantum to define membership, adopting a cultural identity–specific approach rather than a genetic one could allow AIAN populations to move away from an artificially imposed racial past. No other race has been required to prove their identity in this way. The US is becoming more racially/ethnically diverse, including more multiracial partnerships. A Pew Research Center survey^15^ found that most multiracial adults (60%) are proud of their heritage and 59% believe their heritage has made them more open to other cultures. Surveys on race typically include a “more than 1 race” category. This approach may allow for more accurate characterization of race in the US and may help us understand racial differences among those entering medicine.

The American Medical College Application System,^16^ which houses applicant and matriculant data collected by the Association of American Medical Colleges (AAMC), changed how it collected race data in 2002. Before 2002, individuals could only select 1 racial identity. After 2002, the AAMC allowed individuals to select more than 1 race and/or ethnic group on applications. The decision to change may have been attributable to differing trends in applicants, matriculants, and graduates of MD-granting medical schools in the US. The objective of this study was to evaluate how this change in American Medical College Application System data collection in 2002 is associated with temporal trends in applicants, matriculants, and graduates who self-reported as AIAN. These findings could inform diversity efforts.

## Methods

### Data Source and Management

In spring 2018, we purchased a data file from the AAMC that included figures on self-identification as AIAN only and AIAN in combination with other races by medical school applicants (from the American Medical College Application System) and graduates (from the AAMC’s Student Records System) between January 1, 1996, and December 31, 2017. We excluded medical school applicants, matriculants, and graduates who self-identified as non-AIAN races/ethnicities during the same period. We worked closely with AAMC’s data team to resolve inconsistencies when preparing data for analyses. As part of this work, we identified 147 of 8508 persons (1.7%) in the applicant data set who selected different race categories at different times in their applications. These individuals were excluded from the analyses. Only 1 record was used to count graduates; however, not unexpectedly, multiple records existed for applicants because students can apply to many medical schools in multiple years. More than 1 matriculation can also occur because students can be accepted to different medical schools in different years. Study activities were reviewed by Oregon Health & Science University’s institutional review board, and because this was a database study, the board determined that it did not constitute human subjects research and informed consent was not required. All data were deidentified. This study followed the Strengthening the Reporting of Observational Studies in Epidemiology (STROBE) reporting guideline for cohort studies.

### Primary Outcome Variables

The primary outcome variables included yearly rates of applicants, matriculants, and graduates who self-reported as AIAN between January 1, 1996, and December 31, 2017. We divided the period into before and after the American Medical College Application System data collection method change from individuals selecting 1 race and/or ethnic group to having the option to select more than 1 group. For example, during the 1996 to 2001 application cycles, the only available option for individuals who identified as AIAN was selection of AIAN only, even if they were both AIAN and belonged to another racial or ethnic group, such as White or Black. In the 2002-2017 period, applicants could select more than 1 racial and/or ethnic identity, including AIAN with 1 or more other races/ethnicities, demonstrating they identified as multiracial.

### Statistical Analysis

Descriptive statistics, including numbers (percentages) and means (SDs), were used to characterize applicants, matriculants, and graduates. Specifically, we provide descriptive statistics for age, sex, and Medical College Admission Test (MCAT) scores of study participants overall and by period (before and after the data collection change). Differences in characteristics between periods were assessed using a 2-tailed, independent-samples *t* test for continuous variables and a χ^2^ test for categorical variables. We assessed the association between the 2002 change and number of AIAN applicants, matriculants, and graduates separately using interrupted time series (ITS) analyses.^17^ We used ITS regression Poisson models or ITS regression negative binomial models^18^ to estimate the rates of AIAN applicants, matriculants, and graduates immediately after the change in data collection (termed *immediate change*) and the change in trend after the survey change (termed *change in trend*). For the outcome of rates of AIAN applicants, we used ITS negative binomial regression to address observed overdispersion in the outcome. For outcomes of AIAN matriculants and graduates, we used Poisson ITS models because overdispersion was not present for those. For all models, we controlled for age, sex, and MCAT score. Relative rates estimating the immediate change and long-term change of the modification in the survey question are reported.

To visually describe the changes before and after survey modification, we plotted ITS model-based estimates and counterfactual estimates (eg, what we might have expected to see if the race question had not changed) at each year. For all estimates of the rates of AIAN applicants, matriculants, and graduates, we produced 95% prediction intervals to depict uncertainty in our estimates. Statistical testing was 2-sided with type I error set to 5%. All analyses were completed using R, version 3.5.3 (R Foundation for Statistical Computing).

## Results

**Figure 1.**
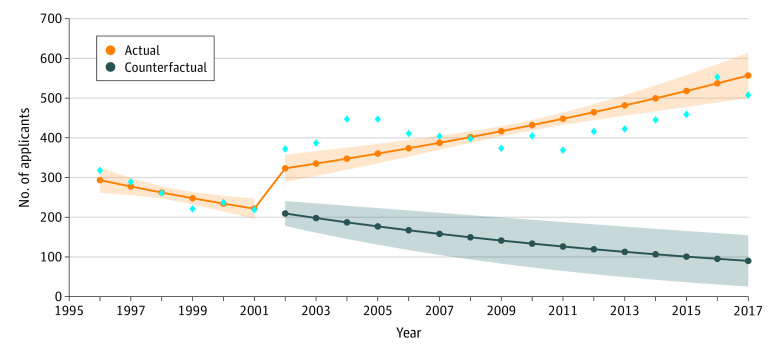
Number of Applicants From 1996-2017 Blue diamonds indicate the observed raw counts for the respective years for applicants. The shading around the orange line indicates 95% CIs, and the shading around the blue line indicates 95% prediction intervals.

The mean (SD) number of applicants per year identifying as AIAN was 380.0 (89.9) overall; there were 257.3 (39.6) from 1996 to 2001 (mean [SD] age of 26.6 [5.5] years; 830 [54.0%] male), and 426.1 (50.1) from 2002 to 2017 (mean [SD] age of 25.5 [5.6] years; 3441 [50.5%] female) (Table 1 and Figure 1). The mean (SD) numbers of matriculants (161.3 [34.8]) and graduates (138.8 [28.9]) over time are also given in Table 1.

**Table 1.** Characteristics of American Indian/Alaska Native Applicants, Matriculants, and Graduates According to Period

Characteristic	Overall (both groups combined)	1996-2001 (AIAN only selected)	2002-2017 (AIAN only and multiple race selected)	*P* value^a^
Age, mean (SD), y	25.8 (5.0)	26.6 (5.5)	25.6 (4.8)	<.001
MCAT score, mean (SD)^b^	25.2 (5.6)	23.8 (5.4)	25.5 (5.6)	<.001
Male sex, No. (%)	4203 (50)	830 (54)	3373 (49.5)	.007
Rate, No. (%)				
Applicants	380.0 (89.9)	257.3 (39.6)	426.1 (50.1)	<.001
Matriculants	161.3 (34.8)	112.0 (13.3)	179.8 (17.4)	<.001
Graduates^c^	138.8 (28.9)	102.3 (11.4)	155.7 (15.0)	<.001

Abbreviations: AIAN, American Indian/Alaska Native; MCAT, Medical College Admission Test.

^a^ Differences between periods were assessed with the independent-samples *t* test, except for number of male individuals, for which the χ^2^ test was used.

^b^ MCAT scores from 2016 to 2017 were standardized to fit the scale of AIAN MCAT scores from 1996 to 2015.

^c^ Excludes the years 2015 to 2017.

The model parameter estimates (ie, baseline counts in 1996, trend before the change in race survey question, and immediate change and change in trend after the modification in the survey question), 95% CIs, and *P* values from ITS model estimating yearly number of AIAN applicants, matriculants, and graduates during all 22 study years (1996-2017) are given in Table 2.

**Table 2.** Adjusted Relative Rates From the Interrupted Time Series Poisson or Negative Binomial Regression Model Estimating Yearly Number of American Indian/Alaska Native Applicants, Matriculants, and Graduates During 22 Years (1996-2017)

Measure	Adjusted relative rate (95% CI)	*P* value^a^
**Applicants**		
Baseline count in 1996	311.88 (271.86-357.78)	<.001
Trend before the change in race data collection	0.95 (0.91-0.98)	.004
Immediate change after the modification in the survey question	1.78 (1.55-2.06)	<.001
Change in trend after the modification in the survey question	1.10 (1.06-1.14)	<.001
Male sex^b^	1.01 (0.99-1.02)	.29
Age^b^	0.96 (0.85-1.08)	.50
MCAT score^b^	0.86 (0.79-0.93)	<.001
**Matriculants**		
Baseline count in 1996	123.77 (102.91-148.86)	<.001
Trend before the change in race data collection	0.97 (0.92-1.02)	.27
Immediate change after the modification in the survey question	1.62 (1.35-1.95)	<.001
Change in trend after the modification in the survey question	1.04 (0.99-1.09)	.09
Male sex^b^	1.01 (0.99-1.02)	.50
Age^b^	0.97 (0.83-1.13)	.69
MCAT score^b^	0.97 (0.88-1.08)	.59
**Graduates**^**c**^		
Baseline count in 1996	112.15 (91.53-137.42)	<.001
Trend before the change in race data collection	0.98 (0.92-1.03)	.38
Immediate change after the modification in the survey question	1.94 (1.57-2.38)	<.001
Change in trend after the modification in the survey question	1.00 (0.95-1.06)	.92
Male sex^b^	1.01 (0.99-1.03)	.33
Age^b^	1.06 (0.89-1.25)	.51
MCAT score^b^	1.05 (0.91-1.22)	.51

Abbreviation: MCAT, Medical College Admission Test.

^a^ Wald tests.

^b^ Variables are mean centered.

^c^ Excludes years 2015 to 2017.

The model estimated the baseline count for the number of applicants to medical school to be 311.88 in 1996 (Table 2). Before the change in data collection (1996-2001), there was a decrease of 5% per year (relative rate [RR], 0.95; 95% CI, 0.91-0.98; *P* < .001) in the mean number of AIAN applicants. In 2002, the change was associated with an immediate 78% relative increase among applicants (RR, 1.78; 95% CI, 1.55-2.06; *P* < .001). In the yearly trend, there was an increase of approximately 10% per year after the survey question modification (RR, 1.10; 95% CI, 1.06-1.14; *P* < .001).

**Figure 2.**
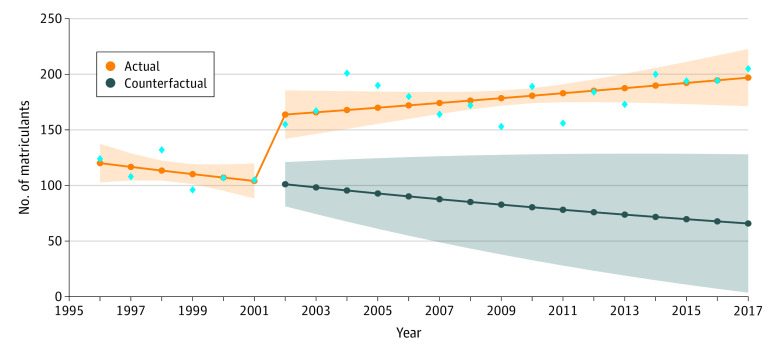
Number of Matriculants From 1996-2017 Blue diamonds indicate the observed raw counts for the respective years for applicants. The shading around the orange line indicates 95% CIs, and the shading around the blue line indicates 95% prediction intervals.

For matriculants to medical school, estimates from the ITS model indicate that before the change in race data collection, there were a mean of 123.77 (95% CI, 102.91-148.86) matriculants in the baseline year of 1996 (Table 2 and Figure 2), with the yearly trend indicating a nonsignificant decrease of 3% (RR, 0.97; 95% CI, 0.92-1.02; *P* = .27) in the mean number of AIAN matriculants before the data collection change. The change was associated with an immediate 62% relative increase in matriculants (RR, 1.62; 95% CI, 1.35-1.95; *P* < .001), with no difference in trend after the change (RR, 1.04; 95% CI, 0.99-1.09; *P* = .09).

**Figure 3.**
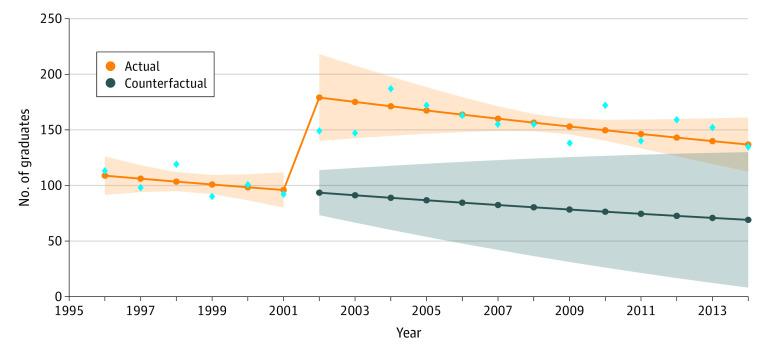
Number of Graduates From 1996-2014 Blue diamonds indicate the observed raw counts for the respective years for applicants. The shading around the orange line indicates 95% CIs, and the shading around the blue line indicates 95% prediction intervals.

Table 2 also gives the mean number of AIAN medical school graduates per year from 1996 to 2014. Interrupted time series modeling estimated that a mean of 112.15 graduates existed in 1996, with a minimal, nonsignificant, yearly trend decrease of 2% (RR, 0.98; 95% CI, 0.92-1.03; *P* = .38) in the mean number of AIAN graduates before the survey change (also see Figure 3). The period after the race/ethnicity question changed is associated with an immediate 94% relative increase in the number of AIAN graduates (RR, 1.94; 95% CI, 1.57-2.38; *P* < .001). The changes in trend from before to after the change in the survey question were not significant (RR, 1.00; 95% CI, 0.95-1.06; *P* = .92).

## Discussion

This cohort study is novel in its focus on how changing data collection for racial/ethnic identity is associated with the measurement of AIAN representation among medical school applicants, matriculants, and graduates from MD-granting medical schools in the US. Issues of racial/ethnic inequities and systemic biases are becoming increasingly important globally and should be considered in educational processes. This study found that when the response options only allowed AIAN for those self-identifying as AIAN, the numbers of applicants, matriculants, and graduates were all lower compared with when individuals could report more than 1 racial/ethnic group. In addition, findings from the counterfactual scenarios indicate downward trends across all 3 outcomes (applicants, matriculants, and graduates) when the race question included AIAN only.

When data collection changed to allow more than 1 racial and/or ethnic group in addition to AIAN, significant increases in all 3 variables were noted immediately after the change, with a significant increasing trend in applicants, a nonsignificant increasing trend in matriculants, and a nonsignificant decreasing trend for graduates. This information is important if programs designed to support AIAN people along medical education pathways are to accurately measure participant outcomes associated with their efforts. Data representing White graduates between 2014 and 2018 note stability in that population during this period.^19^ The current study also examined MCAT scores between AIAN and Whites in existing publications^20^ and found that the mean MCAT score in AIAN populations was 500 compared with means of 507 for Whites and 506 overall in 2018, illustrating only minor differences. Although other studies^21,22^ have assessed applicants and matriculants using either of the 2 periods, this is the first study, to our knowledge, to specifically examine how changing data collection of race/ethnicity is associated with AIAN representation.

The sudden increase in the number of AIAN students identifying as AIAN only or in combination with another race/ethnicity leaves many questions about racial classification and its implications. A decrease in the number of AIAN students identifying as AIAN only is seen, but it is unclear whether students who would previously have identified as AIAN only are now identifying as more than 1 race or whether there truly is a decrease in the number of AIAN-only students applying to medical school. Applicants may choose to convey their race differently for many reasons. It could be for legal/biological reasons or for sociocultural reasons related to shared sacred traditions, languages, places, and history as Indigenous people.^23,24^ Interestingly, some students changed their racial/ethnic identity between applications. Efforts were made to reduce the likelihood of this measurement error by resolving inconsistencies in records when applicants changed their race/ethnicity, and all but 147 of these cases were resolved.

It is unclear how national trends in multiracial partnerships are affecting how AIAN people choose to self-identify race/ethnicity in demographic surveys. The apparent decreasing trend of those who identify as AIAN only may be a result of shifting social norms and one’s concept of self and their decision to identify, or not, with all components of their racial composition. Of note, however, is that the 2010 US census reported 5.2 million people identified as AIAN alone or in combination with 1 or more other races, whereas 2.9 million indicated they were AIAN alone (55.8%).^25^ The 2000 US Census noted that 4.1 million reported being AIAN alone or in combination with 1 or more other races, whereas 2.5 million reported being AIAN only (51.2%).^26^ These data suggest an overall national trend of increased reporting of AIAN.

It was concerning to see the decrease of AIAN-only or AIAN in combination in graduation rates, although this finding was not statistically significant. Many equity programs support AIAN students’ and other underrepresented minority students’ entry into medical school; however, this support may not be enough because studies have found that underrepresented minority students need different support systems during medical school.^27^

There is much to be learned about partnering with tribal nations, schools, and organizations to provide culturally responsive messaging and positive role models for tribal youth so they are optimally supported to become physicians. There are unique challenges in analyzing the AIAN data because of small sample sizes. For example, knowing the high schools that the applicants, matriculants, and graduates attended might help determine what could be learned about these programs and curricula. Similarly, knowledge of tribal affiliations might also have helped. Unfortunately, these data have very small cell sizes and individuals may be identifiable; thus, this study was unable to explore these issues.

Self-identification allows for people to declare their racial designation; however, citizen or descendant status of a federally recognized Indian Nation is a political designation that is determined by each tribe separately. There is limited utility in the disaggregation of *American Indian* and *Alaska Native*, given the uniquely shared political designations of these groups in the US Constitution. Self-identification does not account for this unique political designation or for one’s ability to participate in tribal affairs. It is likely that racial self-identification on a survey is difficult to correlate with tribal, civic, and cultural engagement, and it is becoming more difficult to verify racial status using survey questions alone.^28,29,30^

Tribal citizenship status and self-identification are hotly debated topics^31^; both are fraught with emotion because of long-standing and persistent effects of colonization and oppression.^32^ We recommend that medical schools seek out guidance and voices of local AIAN people, including tribal leaders and lay community members, as well as faculty in the medical school admissions process to help navigate the complexities of AIAN identity. Oregon Health & Science University (OHSU) sought tribal guidance and partnership through the Northwest Portland Area Indian Health Board, which is owned and operated by the 43 regional tribes, before developing the Northwest Native American Center of Excellence. Our research team is led by and primarily composed of American Indians, and we value the expertise and wisdom of tribal communities. Tribal people and communities will continue to inform the complex issues of AIAN identity over time, a journey likely to last decades. Tribal advice has guided the Northwest Native American Center of Excellence and the OHSU School of Medicine Admissions Committee to (1) extend in-state status to AIAN applicants whose tribal homelands are within present-day Oregon, (2) include tribal citizenship on the secondary application, (3) ensure the admissions committee includes AIAN people, and (4) expand AIAN applicant screening to AIAN faculty, staff, and community members. The OHSU has been advised to establish formal government-to-government relationships with regional tribes; these discussions are ongoing with OHSU executive leadership.

### Strengths and Limitations

This study has strengths and limitations. Strengths include that there were more than 100 observations per annual time point and that a large data set that spanned 22 years was used. Efforts were made to reduce measurement error as much as possible, although its complete removal cannot be guaranteed. Other limitations include that the period 1996 to 2001 only contained 6 time points, which subjectively may or may not be enough to evaluate the preperiod trend, especially because it appears that the trend is decreasing. In addition, the study only included data from US MD-granting medical schools, which may not be generalizable to other nations or osteopathic medical schools.

## Conclusions

Changing the way race/ethnicity information was collected resulted in identification of more AIAN applicants, matriculants, and graduates of US MD-granting medical schools and may have affected other race/ethnicity representation as well. Both MD-granting institutions and the AAMC ought not be falsely reassured by the apparent increase of AIAN people in medical education. This increase may be an overrepresentation of AIAN presence because identification as AIAN is a complex matter that has psychological, cultural, and political implications. Data that accurately reflect AIAN representation in MD-granting institutions are important for pathway programs to evaluate their efforts to recruit, train, and retain AIAN physicians. More research is needed to understand how best to improve data collection of AIAN representation in medical school and the physician workforce. Such research will enable medical schools to more effectively leverage resources to best support the journeys of AIAN people who aspire to be physicians.
